# Age-Related Electrocardiographic Characteristics of Male Junior Soccer Athletes

**DOI:** 10.3389/fcvm.2021.784170

**Published:** 2022-02-03

**Authors:** Elena Cavarretta, Luigi Sciarra, Giuseppe Biondi-Zoccai, Francesco Maffessanti, Antonia Nigro, Fabio Sperandii, Emanuele Guerra, Federico Quaranta, Chiara Fossati, Mariangela Peruzzi, Annachiara Pingitore, Dimitrios M. Stasinopoulos, Robert A. Rigby, Rachele Adorisio, Andrea Saglietto, Leonardo Calò, Giacomo Frati, Fabio Pigozzi

**Affiliations:** ^1^Department of Medical-Surgical Sciences and Biotechnologies, Sapienza University of Rome, Latina, Italy; ^2^Mediterranea Cardiocentro, Naples, Italy; ^3^Department of Clinical Medicine, Public Health, Life and Environment Sciences, L'Aquila University, L'Aquila, Italy; ^4^Maria Cecilia Hospital, GVM Care & Research, Cotignola, Italy; ^5^Villa Stuart Sport Clinic, FIFA Medical Centre of Excellence, Rome, Italy; ^6^Department of Movement, Human and Health Sciences, University of Rome “Foro Italico”, Rome, Italy; ^7^London Metropolitan University, London, United Kingdom; ^8^Department of Pediatric Cardiology and Cardiac Surgery, Bambino Gesù Children's Hospital, IRCCS, Rome, Italy; ^9^Division of Cardiology, Department of Medical Sciences, “Città della Salute e della Scienza di Torino” Hospital, University of Turin, Turin, Italy; ^10^Division of Cardiology, Policlinico Casilino, Rome, Italy; ^11^IRCCS Neuromed, Pozzilli, Italy

**Keywords:** athlete's heart, electrocardiogram, adolescent, reference values, exercise, normal values, nomograms

## Abstract

**Introduction:**

Very limited data exist on normal age-related ECG variations in adolescents and no data have been published regarding the ECG anomalies induced by intensive training, which are relevant in pre-participation screening for sudden cardiac death prevention in the adolescent athletic population. The purpose of this study was to establish normal age-related electrocardiographic measurements (P wave duration, PR interval, QRS duration, QT, and QTc interval) grouped according to 2-year age intervals.

**Methods:**

A total of 2,151 consecutive healthy adolescent Soccer athletes (trained for a mean of 7.2 ± 1.1 h per week, 100% male Caucasians, mean age 12.4 ± 1.4 years, range 7–18) underwent pre-participation screening, which included ECG and transthoracic echocardiography in a single referral center.

**Results:**

Their heart rate progressively slowed as age increased (*p* < 0.001, ranging from 80.8 ± 13.2 to 59.5 ± 10.2 bpm), as expected. The P wave, PR interval, and QRS duration significantly increased in older age classes (*p* = 0.019, *p* = 0.001, and *p* < 0.001, respectively), and after Bonferroni's correction, the difference remained significant in all age classes for QRS duration. The QTc interval diminished progressively with increasing age (*p* = 0.003) while the QT interval increased progressively (*p* < 0.001).

**Conclusions:**

Significant variations in the normal ECG characteristics of young athletes exist between different age groups related to increasing age and training burden, thus, age-specific reference values could be adopted, as already done for echocardiographic measurements, and may help to further discriminate potentially pathologic conditions.

## Introduction

The morphological, electrophysiologic, and functional adaptations to regular and intensive physical activity are generally referred to as “the athlete's heart.” Race, sex, type of sport, body size, and age at the start of training significantly impact the characteristics of the adult athlete's heart (1–3), but the pediatric athlete's heart has been characterized less and is usually perceived as influenced to a minor extent by exercise-induced remodeling (4). Nonetheless, during adolescence, there is already a growing level of competitiveness, professionalism, and intensive training that affect this maturation period, such that the International Olympic Committee (IOC) has advanced recommendations to promote the healthy and balanced development of the young athletes (5). A recent meta-analysis (6) has shown that pediatric athletes have a greater prevalence of training-related and training-unrelated ECG changes than non-athletes, and the magnitude, prevalence, and distribution of such changes are dependent on the chronological age of the pediatric athlete. Limited data (7) exist on normal age-related ECG changes in adolescents between different age groups and no data have been published regarding the athletic population classed into age intervals of 2 years. We have already published echocardiographic reference values related to physiological remodeling of the adolescent athlete's heart in soccer players (8), therefore in this study, we aimed to establish normal, age-related electrocardiographic measurements (P wave duration, PR interval, QRS duration, and QT and QTc interval) in a large cohort of adolescent athletes according to age at 2-year intervals, and to provide nomograms to better define the pediatric athlete's heart.

## Methods

### Study Population

This retrospective study included the same healthy population described in detail elsewhere (8). Among the 2,261 subjects initially screened in our center and described in a previous study (9), 2,151 (95%) subjects were included in this study. In total, 110 subjects were excluded from the study because of any abnormal ECG coupled with an abnormal echocardiographic study (11 subjects), an abnormal echocardiographic finding with a normal ECG (91 subjects), or an incomplete study (eight subjects) (10) (Supplementary Figure 1). Briefly, consecutive junior soccer players (7–18 years old, trained for at least 9 months, 100% male, all Caucasian) who underwent pre-participation screening (PPS), including a 12-lead electrocardiogram and transthoracic echocardiography at the Sports Medicine Institute of Rome, Villa Stuart Sport Clinic, FIFA Medical Centre of Excellence, between January 2008 and March 2009 were enrolled. Both the 12-lead ECG and the echocardiographic study were classified as either normal or showing physiological cardiac adaptations to regular exercise (9). Athletes with potential pathological ECGs or echocardiograms have been excluded from this study and analysis. The presence of abnormal or training un-related ECG findings or an abnormal echocardiographic finding, including cardiomyopathy, bicuspid aortic valve, and mitral valve prolapse were considered as the exclusion criteria in order to provide normal values of this population. The local institutional review board approved this retrospective study.

### The 12-Lead Electrocardiogram

All 12-lead resting ECGs were performed using standard equipment (Mortara Instruments, Milwaukee, USA) and were recorded at a paper speed of 25 mm/s and a standard gain of 1 mV/cm. The ECGs were evaluated as previously detailed (10, 11). The heart rate and QRS axis were determined. The P-, Q-, R-, S- and T-wave voltages, ST segments, QRS duration, and PR- and QT-interval were measured with calipers and classified according to the 2017 International Recommendations for electrocardiographic interpretation (12) in athletes as normal, borderline, or abnormal ECG findings. The presence of anterior (V1–V3) T-wave inversion was considered to be a juvenile T-wave pattern in individuals <16 years old in presence of normal echocardiographic findings. The corrected QT interval (QTc) was calculated using the Bazett formula, as already stated (11). Furthermore, borderline ECG findings and a normal echocardiographic study were considered to be evidence of physiological cardiac adaptation to regular exercise and required no further investigation. Two independent sport medicine physicians evaluated the ECGs during PPS. Off-line they also manually measured with the use of calipers the ECGs interval and waves included in the analysis. Moreover, all ECGs were reviewed by a cardiologist who was blinded to the athletes' medical history; discrepancies were resolved after consensus.

### Echocardiography

All athletes underwent a complete transthoracic Doppler echocardiographic study as a part of the PPS, as detailed elsewhere (8).

### Statistical Analysis

Continuous variables are reported as mean ± SD and categorical variables as count (%). Age-wise comparisons were performed with ANOVA and *post-hoc* unpaired *t*-tests with Bonferroni adjustment. Nomogram analysis was performed with the generalized additive models for location, scale, and shape (GAMLSS), which are a general class of statistical models for a univariate response variable developed by Rigby and Stasinopoulos (13). In particular, we relied on the lms function of the GAMLSS package for R (R Foundation for Statistical Computing, Vienna, Austria) and calibrated the centiles, which optimizes the maximum (penalized) likelihood of the model built using the Box-Cox Cole and Green, Box-Cox Power exponential, and Box-Cox t distributions. Two independent experts performed the GAMLSS analysis with dedicated and validated custom-made analyses (DMS changed the GAMLSS functions: calibration, centile.pred, and centiles.boot). Accordingly, centile plots were generated, with accompanying centile tables. For internal validation, we performed bootstrapping, yielding bootstrapped centile tables. Linear correlation was appraised with Pearson correlation, displayed with dendrograms and heatmaps. Statistical significance was set at the 2-tailed 0.05 level. Computations were performed with R 3.6.

## Results

A total of 2,151 male adolescent athletes were included in the analysis and their demographic, anthropometric, and echocardiographic findings are summarized in Table 1. The distribution of HR, P-wave duration, PR interval, QRS duration, and QT and QTc intervals per **2-year** age classes and quartiles are shown in Supplementary Figure 1. We divided it into 2-year classes to be consistent with the previously published studies related to normal ECG systematic values in pediatric patients (7).

**Table 1:** Demographic, anthropometric and echocardiographic measurements of the study population.

**Age (years)**	**Overall**	**Group 1:**	**Group 2:**	**Group 3:**	**Group 4:**	**Group 5:**	**Group 6:**
		**7–8**	**9–10**	**11–12**	**13–14**	**15–16**	**17–18**
***n* (%)**	**2,151 (100)**	**141 (7)**	**448 (21)**	**524 (24)**	**546 (25)**	**383 (18)**	**109 (5)**
Height (cm)	156.9 ± 15.9	132.8 ± 5.8 [2,3,4,5,6]	140.9 ± 7.0 [3,4,5,6]	151.6 ± 8.4 [4,5,6]	165.2 ± 8.9 [5,6]	173.9 ± 7.5 [6]	177.9 ± 6.1
Weight (kg)	49.8 ± 14.8	31.2 ± 6.6 [2,3,4,5,6]	37.1 ± 8.2 [3,4,5,6]	44.6 ± 9.5 [4,5,6]	55.1 ± 10.5 [5,6]	65.3 ± 9.6 [6]	69.6 ± 8.9
Training (hr/week)	7.3 ± 1.2	6.3 ± 0.8 [3,4,5,6]	6.3 ± 0.7 [3,4,5,6]	7.0 ± 1.2 [4,5,6]	8.1 ± 0.7	8.1 ± 0.8	8.2 ± 0.9
Duration of training (months)	29.7 ± 12.7	12.3 ± 1.6 [2,3,4,5,6]	14.5 ± 8.6 [3,4,5,6]	28.4 ± 12.2 [4,5,6]	32.7 ± 16.1 [5,6]	41.4 ± 6.0 [6]	64.3 ± 5.7
SBP (mmHg)	110.0 ± 10.6	103.7 ± 12.0 [3,4,5,6]	105.2 ± 10.7 [3,4,5,6]	108.1 ± 9.4 [4,5,6]	112.6 ± 9.2 [5,6]	114.9 ± 9.4	117.0 ± 7.9
DBP (mmHg)	69.2 ± 7.3	65.6 ± 8.3 [3,4,5,6]	66.7 ± 7.2 [3,4,5,6]	67.9 ± 6.6 [4,5,6]	70.6 ± 6.8 [6]	71.8 ± 6.9	73.3 ± 5.9
LVEDD (mm)	46.1 ± 4.9 (25.0, 58.0)	40.4 ± 3.3 (25.0, 48.0) [2,3,4,5,6]	42.4 ± 3.3 (32.0, 54.0) [3,4,5,6]	44.6 ± 3.6 (33.0, 55.0) [4,5,6]	48.0 ± 3.9 (36.0, 58.0) [5,6]	50.6 ± 3.4 (37.0, 58.0)	51.2 ± 3.7 (35.0, 58.0)
LV Mass (g)	106.1 ± 32.7 (35.4, 234.3)	69.5 ± 15.3 (37.6, 118.2) [2,3,4,5,6]	80.5 ± 17.4 (35.4, 142.9) [3,4,5,6]	94.1 ± 21.6 (41.1, 199.3) [4,5,6]	119.0 ± 27.8 (55.9, 234.3) [5,6]	137.0 ± 24.7 (44.8, 213.9)	142.7 ± 26.9 (51.3, 205.5)

*Data expressed as mean±sd or as absolute count (% of total). SBP, systolic blood pressure; DBP, diastolic blood pressure; LVEDD, left ventricular end-diastolic diameter; LV, left ventricular*.

*p < 0.05 current group vs. the other group, pairwise comparisons with Bonferroni adjustment for multiple comparisons*.

As expected, heart rate progressively slowed with age and increasing hours of training (*P* < 0.001, ranging from 80.8 ± 13.2 to 59.5 ± 10.2 bpm). P wave duration progressively lengthened with age (*P* = 0.019) but significance was lost among the different age classes after Bonferroni correction. PR interval and QRS duration significantly increased in older age classes (*P* = 0.001 and *P* < 0.001, respectively) and after Bonferroni's correction, the difference remained significant in all age classes for QRS duration and groups 1–2 vs. 5–6 for PR interval. QT interval progressively increased over time (*P* < 0.001, Table 1), but this effect was mainly due to heart rate reduction, as proven by the relatively stable QTc interval duration among the different classes. The prevalence of normal or training-related ECG findings was significantly different among age groups (Table 2), with the older age classes displaying the highest prevalence of training-related findings, such as sinus bradycardia, increased QRS voltage for left ventricular hypertrophy, incomplete right bundle branch block, and early repolarization/ST-segment elevation. The prevalence of the anterior T wave inversion reduced progressively with the older age groups, with only 3 (1.3%) cases in 383 athletes aged 15–16 years and 0% in 17–18-year-old athletes, demonstrating the physiological regression of the juvenile pattern with increasing age. Borderline findings were quite uncommon (1.2%) in our series because only athletes with both normal ECGs and echocardiograms were included (Table 3).

**Table 2:** Electrocardiographic measurements per age groups.

**Age (years)**	**Overall**	**Group 1:**	**Group 2:**	**Group 3:**	**Group 4:**	**Group 5:**	**Group 6:**
		**7–8**	**9–10**	**11–12**	**13–14**	**15–16**	**17–18**
***n* (%)**	**2,151 (100)**	**141 (7)**	**448 (21)**	**524 (24)**	**546 (25)**	**383 (18)**	**109 (5)**
HR (bpm)	68.8 ± 12.8 *p* <0.001	80.8 ± 13.2 [54; 109] [2,3,4,5,6]	74.2 ± 12.5 [52; 101] [3,4,5,6]	70.2 ± 11.4 [50; 96] [4,5,6]	66.3 ± 11.3 [47; 93] [5,6]	62.5 ± 11.2 [44; 88]	59.5 ± 10.2 [43; 82]
P dur (msec)	69.2 ± 18.3 *p* = 0.019	66.2 ± 16.6 [34; 93]	68.0 ± 18.6 [35; 93]	68.5 ± 18.0 [37; 94]	69.7 ± 17.9 [39; 95]	71.4 ± 19.0 [42; 97]	71.1 ± 20.3 [44; 99]
PR (msec)	136.7 ± 26.8 *p* = 0.001	132.5 ± 24.8 [101; 178] [5,6]	134.1 ± 26.5 [105; 179] [5,6]	136.0 ± 24.6 [107; 180]	136.5 ± 28.6 [109; 184]	140.6 ± 25.1 [112; 188]	142.7 ± 33.8 [115; 193]
QRS (msec)	90.9 ± 10.4 *p* <0.001	87.4 ± 8.5 [71; 106] [4,5,6]	87.0 ± 9.0 [72; 108] [3,4,5,6]	89.1 ± 10.3 [74; 111] [4,5,6]	91.6 ± 9.8 [76; 113] [5,6]	96.2 ± 10.2 [79; 115]	97.8 ± 9.7 [81; 117]
QT (msec)	377.9 ± 30.0 *p* <0.001	355.9 ± 25.4 [312; 413] [2,3,4,5,6]	367.9 ± 29.8 [319; 424] [3,4,5,6]	375.1 ± 26.2 [325; 434] [4,5,6]	383.1 ± 29.5 [330; 442] [5,6]	389.7 ± 28.6 [332; 448]	393.1 ± 29.5 [333; 452]
QTc (msec)	395.9 ± 23.3 *p* = 0.003	397.2 ± 19.8 [355; 439]	397.5 ± 20.5 [355; 440] [6]	396.9 ± 21.4 [355; 440] [6]	396.4 ± 20.6 [353; 438] [6]	393.6 ± 21.2 [349; 434]	389.9 ± 23.3 [344; 430]

*Data expressed as mean ± sd [2nd; 98th percentile]*.

*^[]^p < 0.05 current group vs. Group J, pairwise comparisons with Bonferroni adjustment for multiple comparisons*.

*HR, heart rate; P dur, P-wave duration*.

**Table 3:** Prevalence of training-related ECG findings or borderline findings in the study population per age group.

**Finding**	**Overall**	**Group 1:**	**Group 2:**	**Group 3:**	**Group 4:**	**Group 5:**	**Group 6:**	***p***
		**7–8**	**9–10**	**11–12**	**13–14**	**15–16**	**17–18**	
	**2,151 (100)**	**141 (7)**	**448 (21)**	**524 (24)**	**546 (25)**	**383 (18)**	**109 (5)**	
Sinus bradycardia (<60 bpm)	512 (23.8%)	9 (6.3%)	41 (9.3%)	84 (15.9%)	152 (27.8%)	167 (43.4%)	59 (53.6%)	<0.001
Increased QRS voltage for LVH	311 (14.5%)	13 (9.2%)	41 (9.3%)	65 (12.3%)	107 (19.6%)	63 (16.4%)	22 (20.0%)	<0.001
Incomplete RBBB	689 (32%)	30 (21.1%)	87 (19.9%)	138 (26.1%)	198 (36.3%)	189 (49.1%)	47 (42.7%)	<0.001
Early repolarization/ST segment elevation	407 (18.9%)	18 (12.7%)	63 (14.4%)	93 (17.6%)	139 (25.5%)	75 (19.5%)	19 (17.3%)	<0.001
Anterior (V1–V3) T wave inversion age <16 years old	120 (5.6%)	21 (14.8%)	49 (11.2%)	32 (6.1%)	14 (2.6%)	3 (1.3%)	0 (0%)	<0.001
Ectopic atrial or junctional rhythm	95 (4.4%)	2 (1.4%)	27 (6.0%)	20 (3.8%)	25 (4.6%)	15 (3.9%)	6 (5.5%)	0.326
1st degree AV block	9 (0.4%)	1 (0.7%)	2 (0.5%)	1 (0.2%)	2 (0.4%)	1 (0.3%)	2 (1.8%)	0.269
Left axis deviation	18 (0.8%)	2 (1.4%)	3 (0.7%)	4 (0.8%)	3 (0.6%)	5 (1.3%)	1 (0.9%)	0.812
Left atrial enlargement	0 (0%)	0 (0%)	0 (0%)	0 (0%)	0 (0%)	0 (0%)	0 (0%)	- -
Right axis deviation	2 (0.1%)	0 (0%)	0 (0%)	0 (0%)	1 (0.2%)	1 (0.3%)	0 (0%)	0.734
Right atrial enlargement	5 (0.2%)	0 (0%)	0 (0%)	1 (0.2%)	2 (0.4%)	2 (0.5%)	0 (0%)	0.633
Complete RBBB	1 (0.1%)	0 (0%)	0 (0%)	0 (0%)	0 (0%)	1 (0.3%)	0 (0%)	0.853

*Data expressed as absolute count (% of total). AV, atrio-ventricular; LVH, left ventricular hypertrophy; RBBB, right bundle branch block*.

*^**[]**^p < 0.05 current group vs. the other group, pairwise comparisons with Bonferroni adjustment for multiple comparisons*.

**Figure 1:**
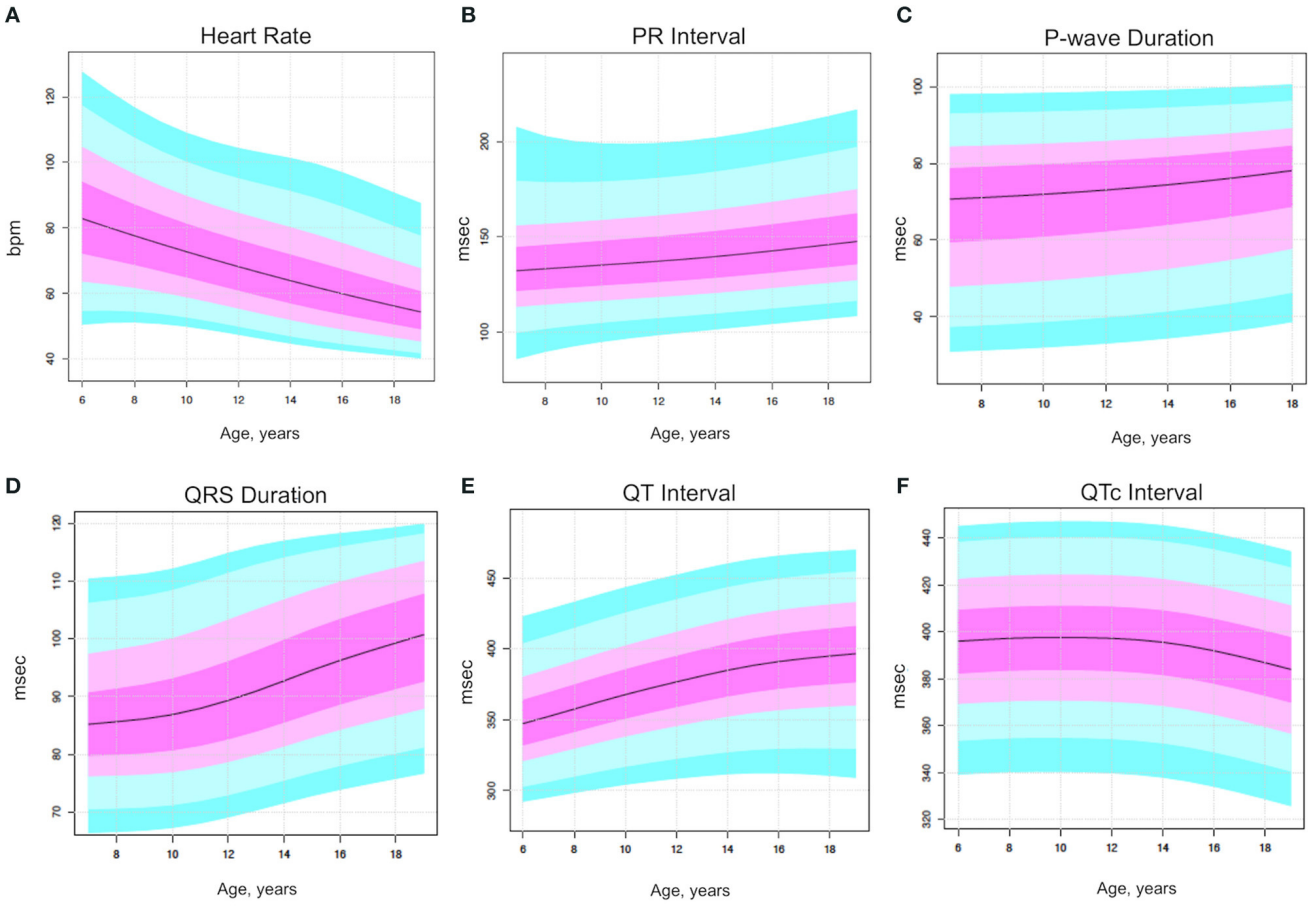
Centile rainbow plots for heart rate (top left panel), P wave duration (right panel), PR (mid-left panel), QRS (mid-right panel), QT (bottom left panel), and QTc (bottom right panel), expressed as median and quartile and age ranges. **(A)** Heart rate. **(B)** *P* wave duration. **(C)** PR duration. **(D)** QRS duration. **(E)** QT duration. **(F)** QTc duration.

**Figure 2:**
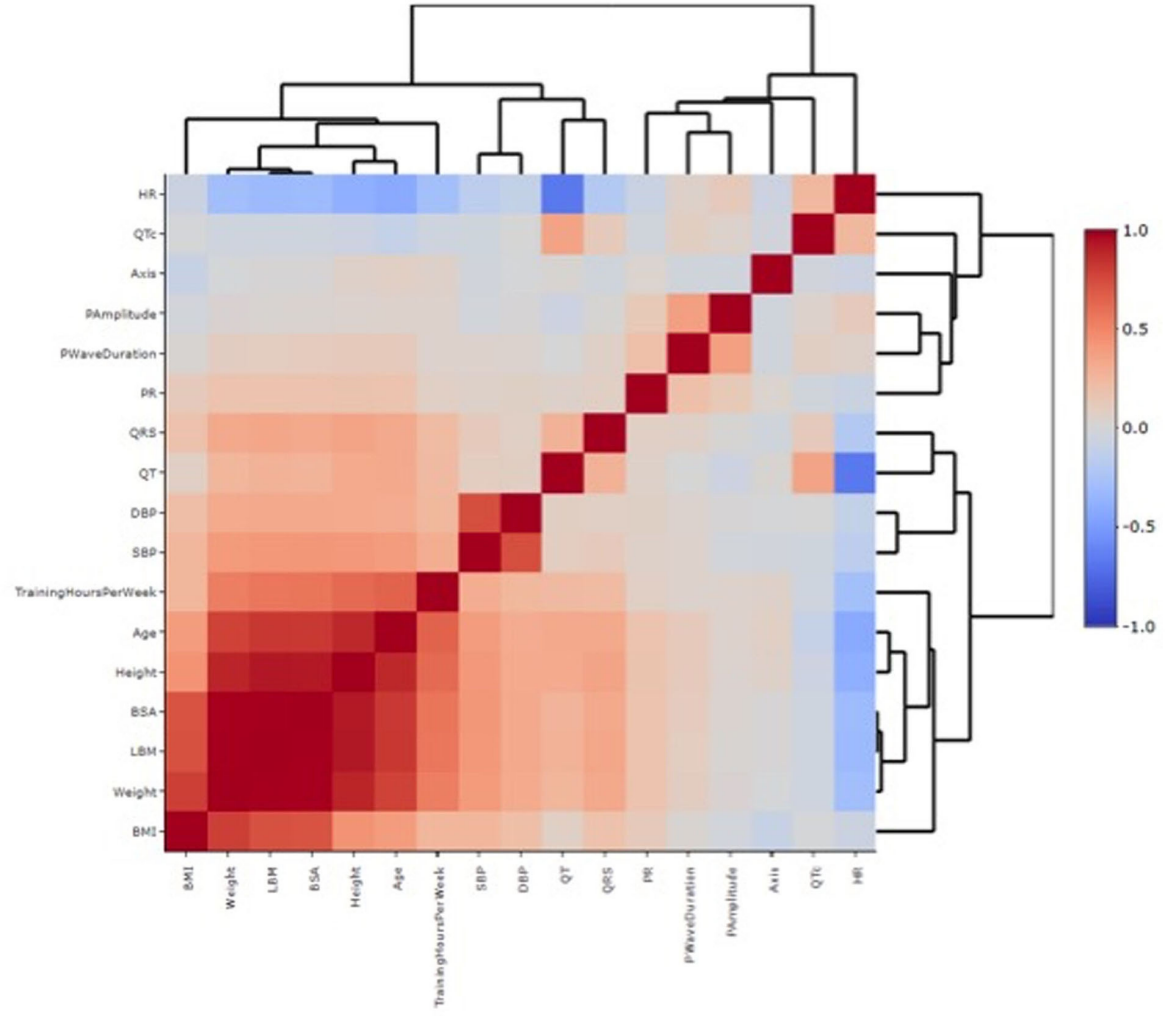
Heatmap correlation between individual features and ECG variables. Each square of the heatmap represents the correlation between the x-axis and the y-axis variables, which ranges between −1 and +1. The closer to +1 the stronger the correlation is (red in the legend, high correlation). The closer to −1 there is an inverse correlation (blue in the legend). White color represents no correlation. The diagonals are all dark red because the heatmap plot is symmetrical about the diagonal and those squares represent the same variable paired together on both axes. The dendrogram highlights the different clusters in which the study population has been divided based on the different variables analyzed.

**Figure 3:**
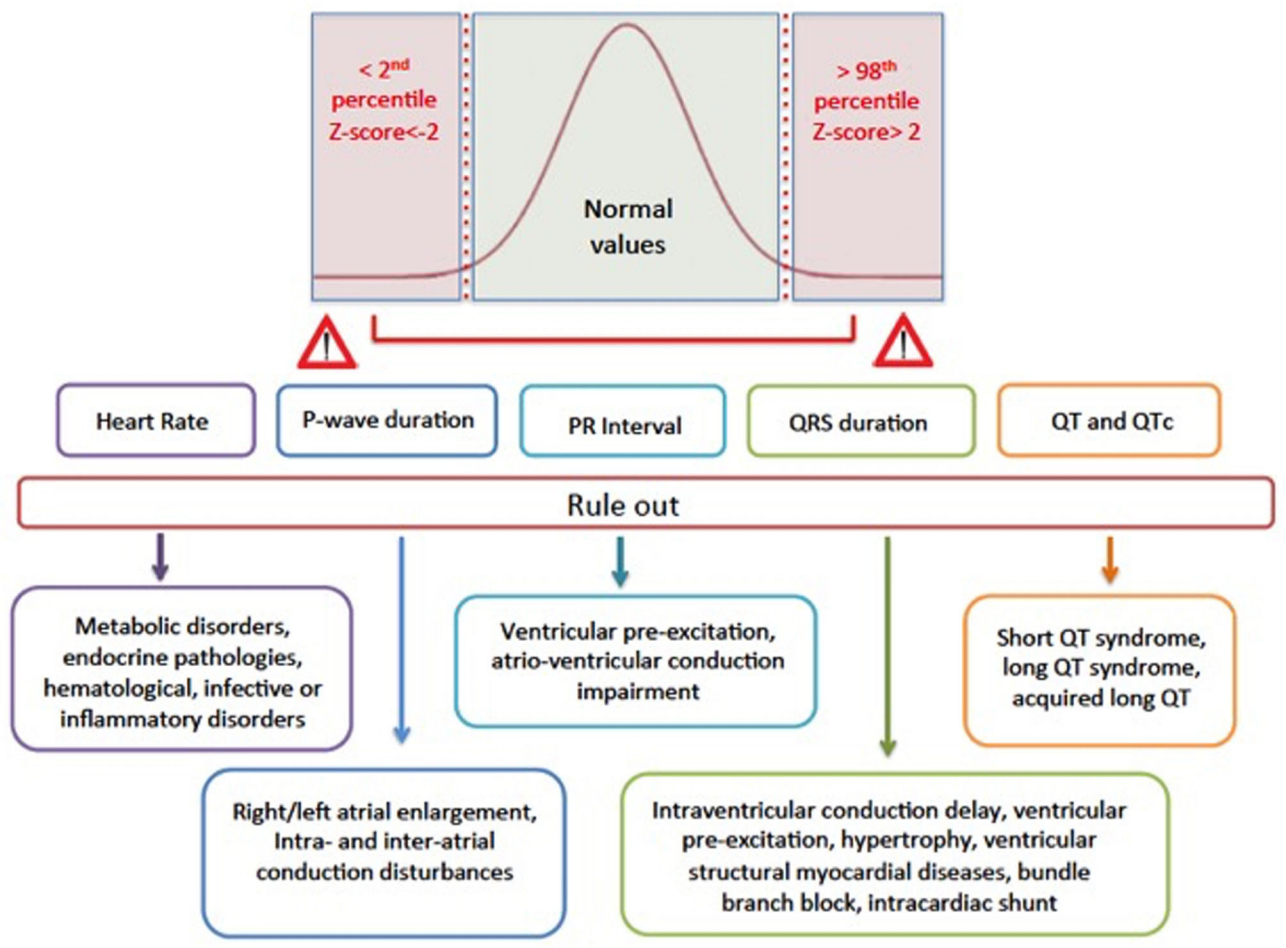
Graphical representation of why reference values are important in clinical practice. Values <2nd or >98th percentiles correspond to *Z*−scores of < -2 or > 2, respectively, and they may imply the presence of a specific pathology or disease that should be ruled out because that value is outside the normal values, present in the 96% of the study population.

The nomogram analysis generated detailed centile plots and centile tables for all the parameters of interest, including HR, P wave duration, PR, QRS, QT, and QTc (Figures 1, 2). Both the figures and the Supplementary Tables may be useful in identifying normal values based on age classes and centiles. The x-axis shows the age in years, while the y-axis indicates the ECG parameter value. The usefulness of using nomograms is depicted in Figure 3.

Correlation analysis highlighted the substantial correlation between the different anthropometric characteristics, arterial pressure measurements, and the ECG parameters, whereas correlations with other features were less robust (Supplementary Table 1; Supplementary Figure 3). In particular, there was a strong correlation between PR, QRS, and QT duration in milliseconds with all the anthropometric characteristics [body mass index [BMI], body surface area (BSA), lean body mass, height, and weight], with age and with hours of training per week. Systolic and diastolic blood pressure values correlated with the anthropometric characteristics and age but not with the ECG parameters. For HR, an inverse correlation was found with age, training hours, and QT, which was not significant when QTc was considered.

## Discussion

Over recent years, several nomograms and reference values for echocardiographic parameters in athletes have been published, including left (LV) and right ventricular dimensions, mass, volumes, and aortic root measurements, but to the best of our knowledge, no nomograms exist for electrocardiographic parameters in adolescent athletes. Obtaining reference values in adolescents is especially demanding due to factors such as the development stage and age, and their complex interplay between puberty, sex, and response to training. ECG is the most commonly used test to screen the pediatric population in competitive sports and its interpretation is based on the knowledge on how ECG parameters modify through growth and according to sex. Age and sex-related differences are summarized from the observation that from birth through adolescence, principal modifications are related to decreasing heart rate, increasing QRS voltages, and a widening QRS complex. These data have been provided for the non-athlete pediatric population (14).

In this study, we have sought to provide reference values for normal ECG parameters in a population of more than 2,000 junior athletes evaluated with ECG and echocardiography, which constitute a unique study population of healthy athletes. Our study has shed light for the first time on the temporal evolution of cardiovascular adaptations and describes common training-related ECG modifications in healthy athletes during adolescence, namely: (1) progressive HR reduction culminating in sinus bradycardia; (2) progressive PR elongation which can lead to 1st degree atrioventricular block; (3) increasing QRS duration and development of an incomplete right bundle branch block, while (4) the QTc remains unchanged over time. As pointed out by Cantinotti et al. (15) in their critical review, the current echocardiographic nomograms are limited by numerical and methodological issues, therefore, to generate our electrocardiographic nomograms we used an appropriate sample size and a rigorous statistical approach.

The increasingly early age at which young talents embark on a professional career path, together with growing competitiveness, means that the practice of sports cardiology needs to adapt to guarantee age-appropriate, comprehensive pediatric cardiac evaluations (16). Intensive training already begins to elicit physiological adaptations in athletes as young as 12 years, this change being more pronounced in endurance athletes (6, 17, 18). However, although the structural and functional cardiovascular adaptations to intense exercise are less pronounced when compared with adult athletes, current guidelines do not reflect this distinction, treating adolescent and adult athletes equally. When evaluating an athlete during childhood or adolescence, it is important not only to rule out high-risk features for sudden cardiac death and distinguish physiological adaptations from pathological remodeling but also to take into consideration somatic growth and pubertal development (19–21).

### ECG Findings in Athletes vs. High-School Students

The current literature has mainly focused on the prevalence and evolution of uncommon and training-unrelated ECG findings, such as T-wave inversion, which are a common feature of the juvenile pattern when present in the anterior leads (9, 22); however, less is known about age-related physiological ECG characteristics in adolescents. Santini et al. (7) evaluated a substantial population of 24,062 high-school students, describing the anthropometric characteristics, baseline ECG findings, and main clinical findings according to age groups at 2-year intervals. The mean HR slowed with increasing age, as in our population, however, HR ranged from 83.6 bpm for 12–13 years to 74.3 bpm for 18–19 years, while in our population of trained athletes, the same age classes manifested a significantly lower HR value (68 bpm for 12–13 years and 59 bpm for 17–18 years), suggesting the significant impact of intense training. Similarly, the PR interval was also increased (133 ms for 12–13-year-old high-school adolescents vs. 136 ms in 12–13-year-old athletes, reaching 143 ms at 18 years of age) as was the QRS duration (85 ms for 12- to 13-year adolescents vs. 90 ms for athletes, reaching 98 ms at 18 years of age), while the QT was inversely related, probably due to HR reduction (7). Only 67% of the study population of high-school students reported exercising regularly and this percentage declined at higher classes of age, dropping to 57% at 18 years, so almost a third of the student population was composed of sedentary adolescents. However, no data specific to competitive athletes were reported, which conversely constitutes our entire study population and may, therefore, explain the significant differences found when compared to our data.

A similar evolution of the QTc interval in athletes over time has been previously described in the literature (23).

### The Usefulness of ECG Nomograms

To the best of our knowledge, the electrocardiographic reference values we have provided represent a precise topography of the physiological evolution of the athlete's heart over time during adolescence and bring to light the growing prevalence of cardiovascular adaptations to intense and regular training in mixed sports athletes, in which hemodynamic responses and long-term impact on cardiac output and remodeling are balanced between dynamic and static components (24). Recently, several articles have been published on the electrocardiographic characteristics of adolescent athletes (25–27), but none have evaluated the evolving difference between 2-year age classes or provided specific reference values in adolescent athletes with a normal ECG and a normal echocardiographic evaluation. We believe that our electrocardiographic nomograms could be extremely useful in defining normal reference limits and z-scores among different groups of age to aid in differentiating training adaptations from normal growth and maturation, which may be of particular importance when an inherited disease is suspected (Figure 3). Sudden cardiac death usually debuts in the young, especially in adolescents affected by inherited arrhythmic diseases and cardiomyopathy. Consequently, improved screening techniques for detecting are strongly required in the clinical arena (28). Recently a large, multicenter study in childhood hypertrophic cardiomyopathy demonstrated that ECG abnormalities were common and varied, but none of them, either in isolation or in the ECG risk score, were associated with the 5-year sudden cardiac death risk (29). Potentially, other individual ECG parameters may improve the current risk prediction models. Bratincsák et al. (14) developed normative standards for 102 ECG variables in the young utilizing z-scores and proposed that expressing ECG variables by *z*-scores will lend to an objective and reproducible evaluation and more confident establishment of ECG-disease correlations. Establishing normative standards and corresponding *z*-scores will be the first step forward a standardized and unbiased approach of assessing ECG variables regardless of age, heading to their use also in sports cardiology. We may speculate that using these reference values and *z*-scores may help to better define the degree of physiological adaptations found in an athlete to support the diagnosis of training-related changes. Referring to both electrocardiographic and echocardiographic nomograms may further enhance the accuracy of PPS in identifying those individuals who fall into a gray zone between the physiological remodeling of the athlete's heart vs. the pathological onset of inherited cardiomyopathy or channelopathy. Appropriate use of second-level investigation such as MRI as discrepancies relative to the reference electrocardiographic and echocardiographic values arise may successfully rule out or identify an underlying pathology.

### Limitations

We must address some study limitations for all retrospective studies. Because of the homogenous study population with respect to sex, ethnicity, and sport practiced (soccer), our results should not be generalized to female adolescent athletes, non-Caucasian athletes, or athletes practicing other sports. Soccer is a mix of high dynamic and moderate static demand; therefore, our results cannot be reliably applied to athletes from power, endurance, or skill disciplines, in particular, our findings may be even more pronounced in endurance athletes. Nevertheless, we are convinced that the novelty of our data can provide an overall idea of the evolution of ECG characteristics among age groups in athletes in general. The effects of training could not be evaluated in a group of matched controls but only with the data available in the literature. The stage of puberty was not accurately assessed using standardized tools, such as the Tanner scale, and we cover a heterogeneous age range from 7 to 18 years. This research should be seen as a preliminary study and the hypothetical additional value of using z-scores in ECG reports needs to be tested in a larger series of adolescents with normal and pathological findings. Such an investigation is currently underway at our centers and the results will be the object of future publications.

## Conclusion

This study has shed light on the physiological adaptations to exercise among different classes of age in a large cohort of healthy junior athletes. Referring to the specific age-related normal values may provide a more specific reading of the adolescent athlete's ECG. We are confident that this data may be helpful in providing a standardized and unbiased analysis of the ECG to better help discriminate between growth- and training-induced changes and pathological remodeling.

## Data Availability Statement

The raw data supporting the conclusions of this article will be made available by the authors, without undue reservation.

## Ethics Statement

The studies involving human participants were reviewed and approved by Sapienza University of Rome. Written informed consent to participate in this study was provided by the participants' legal guardian/next of kin.

## Author Contributions

EC, GB-Z, FM, and LS contributed to the conception or design of the study. AN, FS, EG, FQ, CF, AP, and EC contributed to data acquisition, analysis, and interpretation. EC, GB-Z, RR, and DS performed statistical analyses. EC drafted the manuscript. LS, AN, FM, EG, FQ, CF, RA, AS, GF, LC, FP, MP, AP, RR, DS, GB-Z, and EC critically revised the manuscript for key intellectual content. All the authors gave final approval and agree to be accountable for all aspects of work ensuring integrity and accuracy.

## Funding

The authors received financial support for the research by Sapienza University of Rome, Italy (grant prot. RM11816433B92B68) to EC and by Villa Stuart Sports Clinics, FIFA Medical Center of Excellence.

## Conflict of Interest

Author GB-Z has consulted for Cardionovum, Bonn, Germany; Innovheart, Milan, Italy; Meditrial, Rome, Italy; Replycare, Rome, Italy. The remaining authors declare that the research was conducted in the absence of any commercial or financial relationships that could be construed as a potential conflict of interest.

## Publisher's Note

All claims expressed in this article are solely those of the authors and do not necessarily represent those of their affiliated organizations, or those of the publisher, the editors and the reviewers. Any product that may be evaluated in this article, or claim that may be made by its manufacturer, is not guaranteed or endorsed by the publisher.
