# Supplementary material for: Age-Related Electrocardiographic Characteristics of Male Junior Soccer Athletes
Source: Front Cardiovasc Med. 2022 Feb 3;8:784170. doi: 10.3389/fcvm.2021.784170 (PMC8850359; doi:10.3389/fcvm.2021.784170)

**Online Supplement to CAVARRETTA ET AL. - Age-related electrocardiographic characteristics of male junior Soccer athletes**

Figure 1S. Flow-chart of the study population. Adapted from figure 3 Calò L, Martino A, Tranchita E, Sperandii F, Guerra E, Quaranta F, Parisi A, Nigro A, Sciarra L, Ruvo E, Casasco M, Pigozzi F. Electrocardiographic and echocardiographic evaluation of a large cohort of peri-pubertal soccer players during pre-participation screening. Eur J Prev Cardiol. 2019 Sep;26(13):1444-1455. doi: 10.1177/2047487319826312.


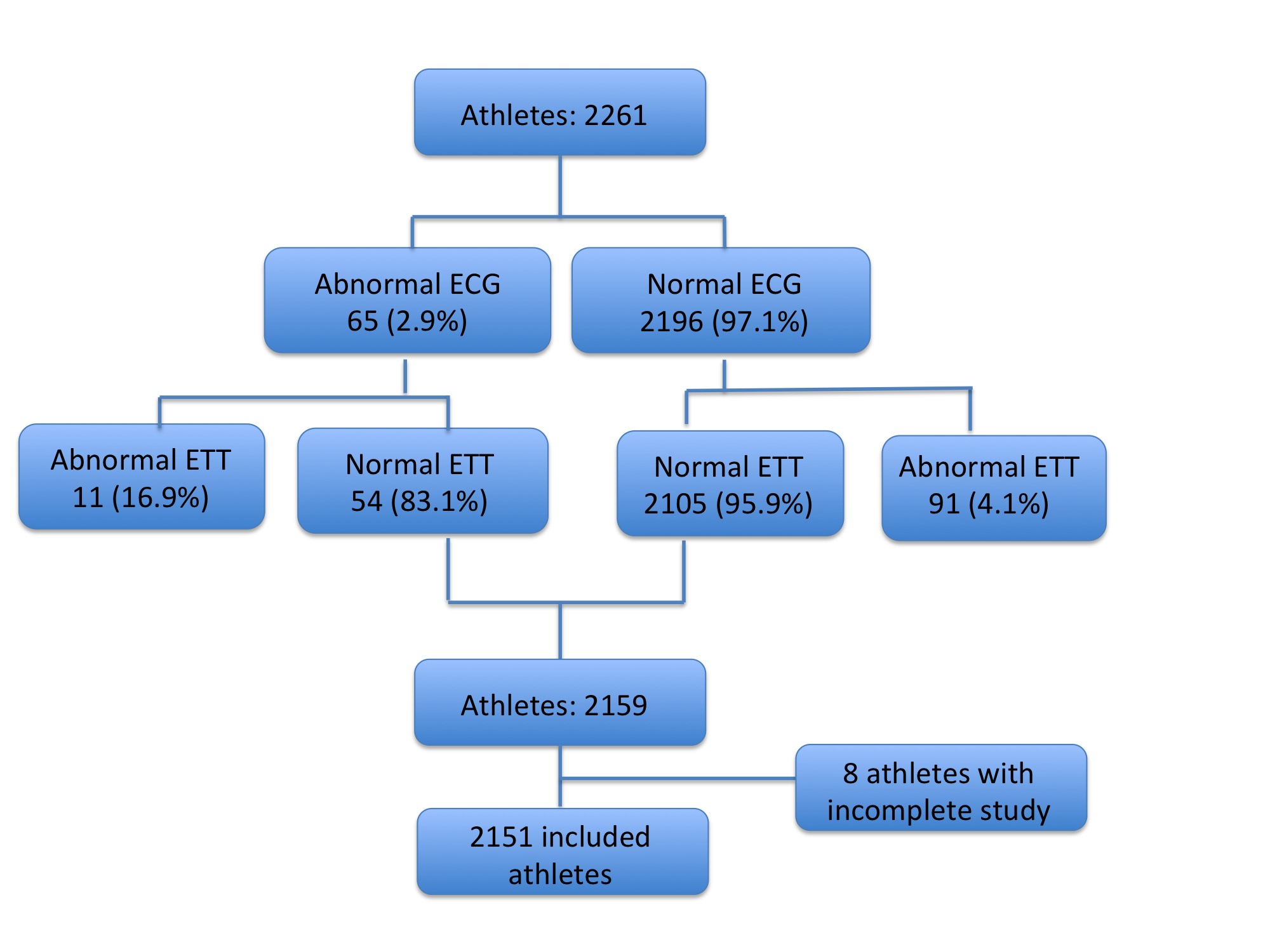


Figure 2 S. Electrocardiographic parameters expressed as median and quartile and age ranges for heart rate (top left panel), P wave duration (to right panel), PR (mid left panel), QRS (mid right panel), QT (bottom left panel), QTc (bottom right panel).

Figure 3S. Scatterplots and superimposed centile line plots for heart rate (top left panel), P wave duration (top left panel), PR interval (top left panel C), QRS duration (top left panel), QT interval (top left panel), QTc interval (top left panel).

**Table 1S.** Pearson correlation coefficient r between elettrocardiographic and demographic or anthropometric measurements.

|  | **Age** | **Weight** | **Height** | **BMI** | **BSA** | **Training** |
| --- | --- | --- | --- | --- | --- | --- |
| **HR** | -0.42 (-0.46,0.38) | -0.29 (-0.33,-0.25) | -0.38 (-0.42,-0.34) | -0.01 (-0.05,0.04) | -0.32 (-0.36,-0.28) | -0.76 (-0.82,-0.67) |
| **P dur** | 0.08 (0.04,0.12) | 0.06 (0.02,0.11) | 0.08 (0.04,0.13) | 0.01 (-0.03,0.06) | 0.07 (0.02,0.11) | 0.14 (0.07,0.22) |
| **PR** | 0.11 (0.06,0.15) | 0.10 (0.06,0.14) | 0.11 (0.06,0.15) | 0.07 (0.03,0.12) | 0.11 (0.07,0.15) | 0.19 (0.12,0.27) |
| **QRS** | 0.32 (0.28,0.36) | 0.33 (0.29,0.37) | 0.35 (0.31,0.39) | 0.19 (0.15,0.23) | 0.35 (0.31,0.39) | 0.59 (0.51,0.66) |
| **QT** | 0.32 (0.28,0.36) | 0.25 (0.21,0.29) | 0.32 (0.28,0.36) | 0.08 (0.04,0.12) | 0.28 (0.24,0.32) | 0.58 (0.51,0.66) |
| **QTc** | -0.09 (-0.13,-0.05) | -0.04 (-0.08,0.00) | -0.05 (-0.09,0.00) | -0.01 (-0.05,0.04) | -0.04 (-0.08,0.00) | -0.17 (-0.24,-0.09) |

**Abbreviations**: HR= heart rate; P dur = P-wave duration; PR = PR interval duration; QRS = QRS duration; QT = QT interval duration; QTc = heart rate-adjusted QT interval.

Figure 4S. Nomogram analysis for heart rate: scatterplot.


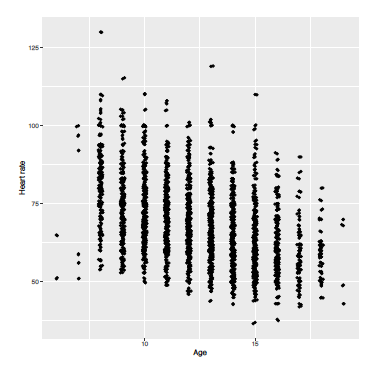


Figure 5S. Nomogram analysis for heart rate: modeling diagnostics.


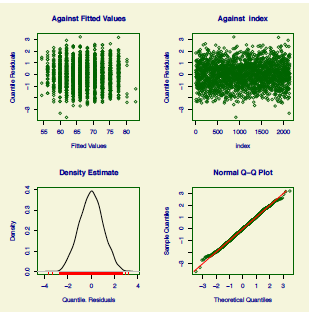


Figure 6S. Nomogram analysis for heart rate: scatterplot and superimposed centile line plot.


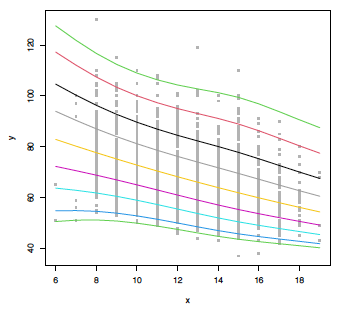


Figure 7S. Nomogram analysis for heart rate: centile rainbow plot.


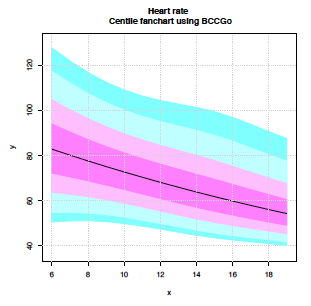


Figure 8S. Nomogram analysis for heart rate: calibrated centile plot


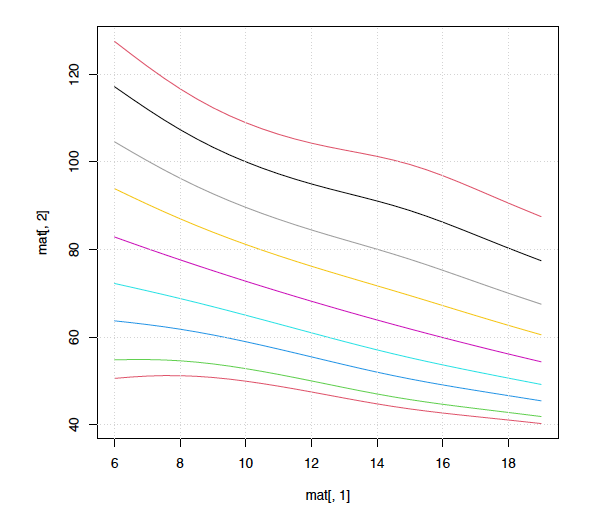


Figure 9S. Nomogram analysis for heart rate: bootstrap 95% confidence intervals for the centiles (2; 5; 98) plot.


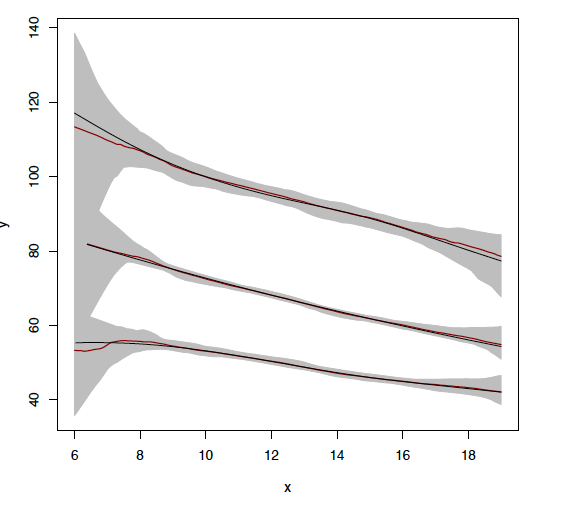


Figure 10S. Nomogram analysis for P wave duration against age: scatterplot.


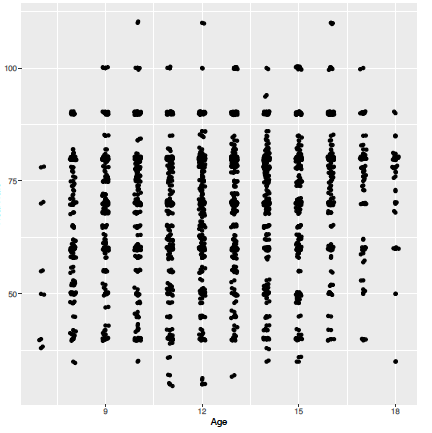


Figure 11S. Nomogram analysis for P wave duration: the residual plot.


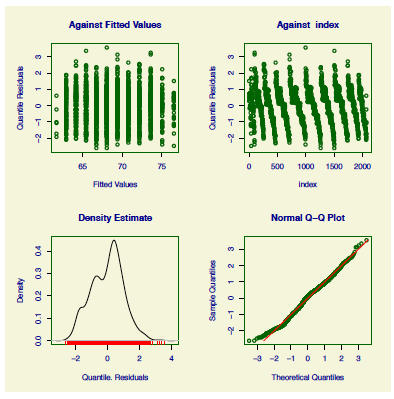


Figure 12S. Nomogram analysis for P wave duration: scatterplot and superimposed centile line plot.


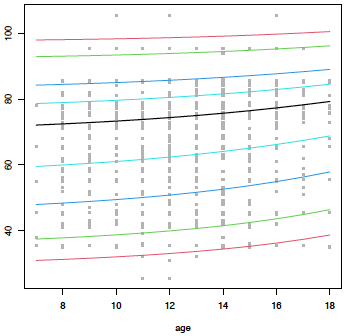


Figure 13S. Nomogram analysis for P wave duration: centile rainbow plot.


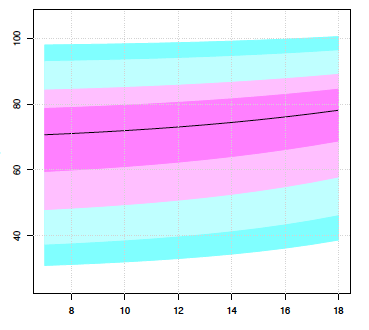


Figure 14S. Nomogram analysis for P wave duration: predicted centile plot.


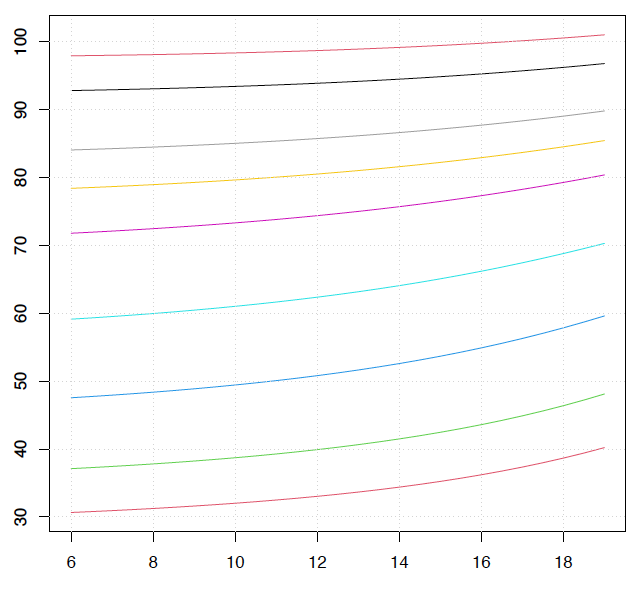


Figure 15S. Nomogram analysis for P wave duration: bootstrap 95% confidence interval for the centiles (2; 50; 98).


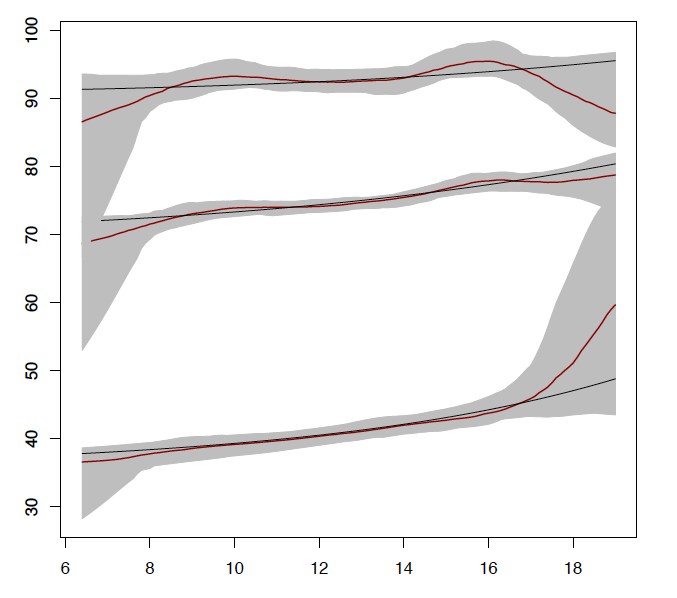


Figure 16S. Nomogram analysis for PR: scatterplot.


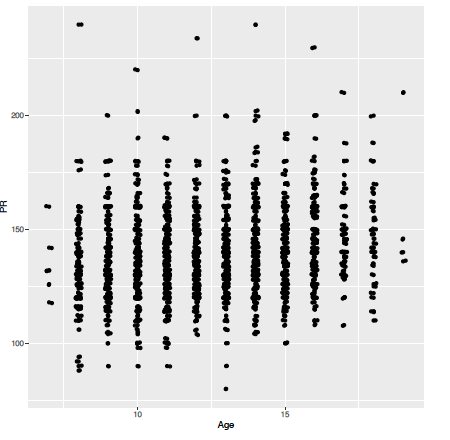


Figure 17S. Nomogram analysis for PR: modeling diagnostics.


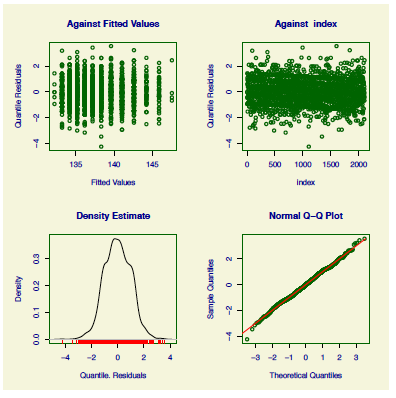


Figure 18S. Nomogram analysis for PR: scatterplot and superimposed centile line plot.


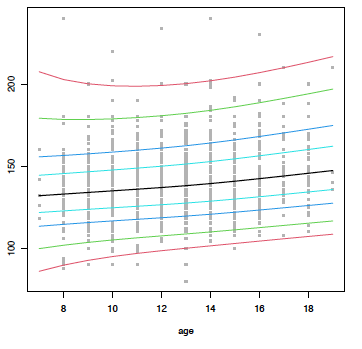


Figure 19S. Nomogram analysis for PR: centile rainbow plot.


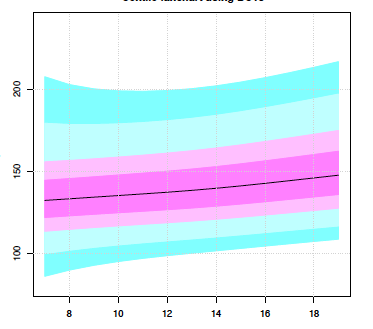


Figure 20S. Nomogram analysis for PR: predicted centile plot.


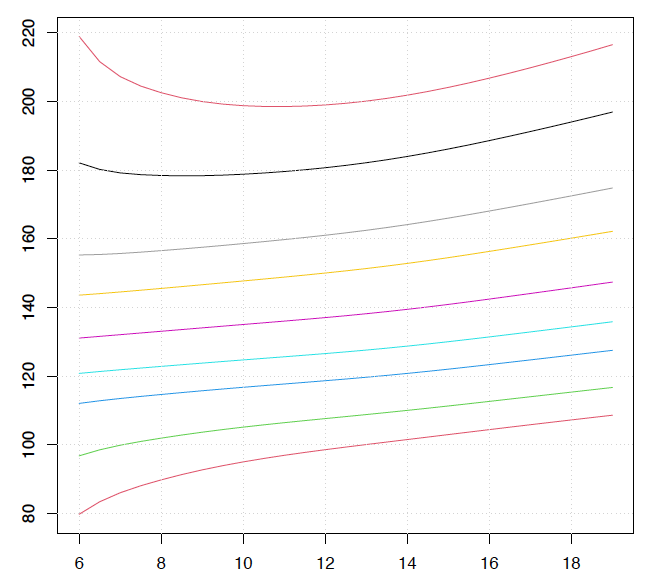


Figure 21S. Nomogram analysis for PR: bootstrap 95% confidence interval for the centiles (2; 50; 98).


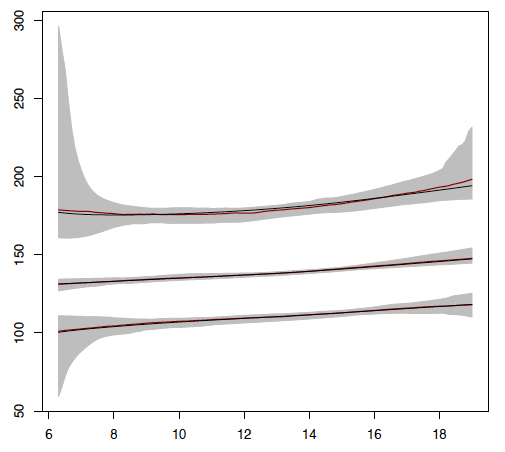


Figure 22S. Nomogram analysis for QRS: scatterplot.


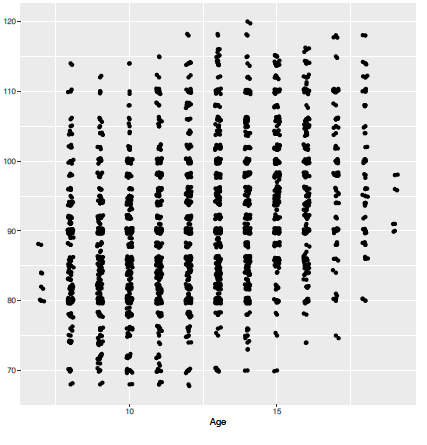


Figure 23S. Nomogram analysis for QRS: modeling diagnostics.


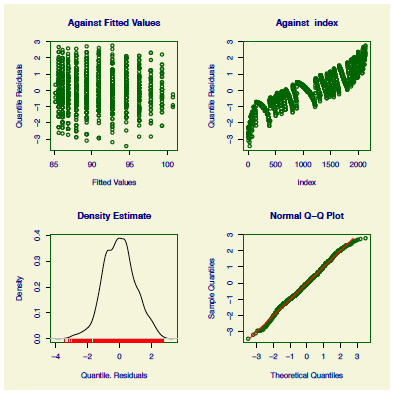


Figure 24S. Nomogram analysis for QRS: scatterplot and superimposed centile line plot.


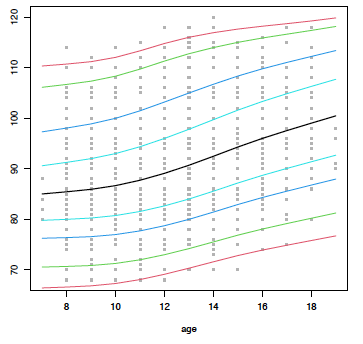


Figure 25S. Nomogram analysis for QRS: centile rainbow plot.


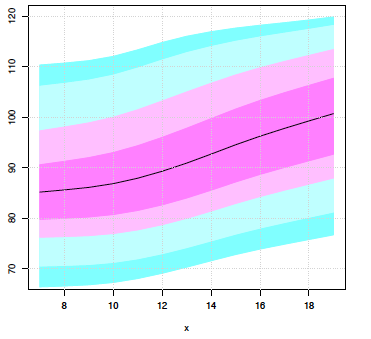


Figure 26S. Nomogram analysis for QRS: predicted centile plot.


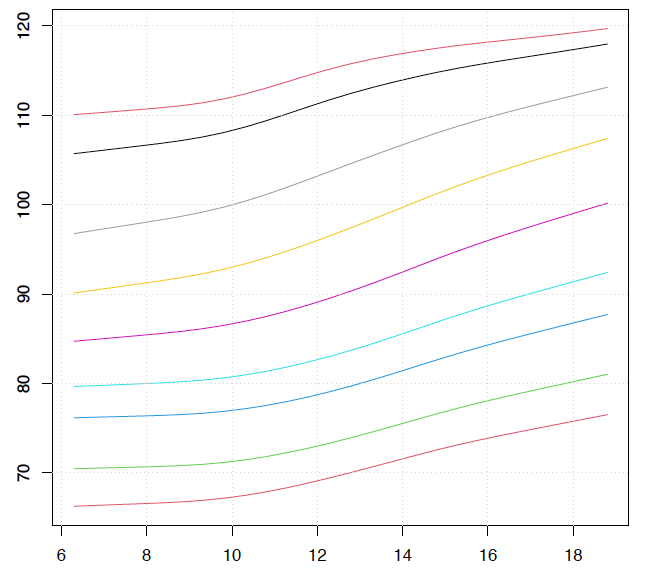


Figure 27S. Nomogram analysis for QRS: bootstrap 95% condience interval for the centiles (2; 50; 98).


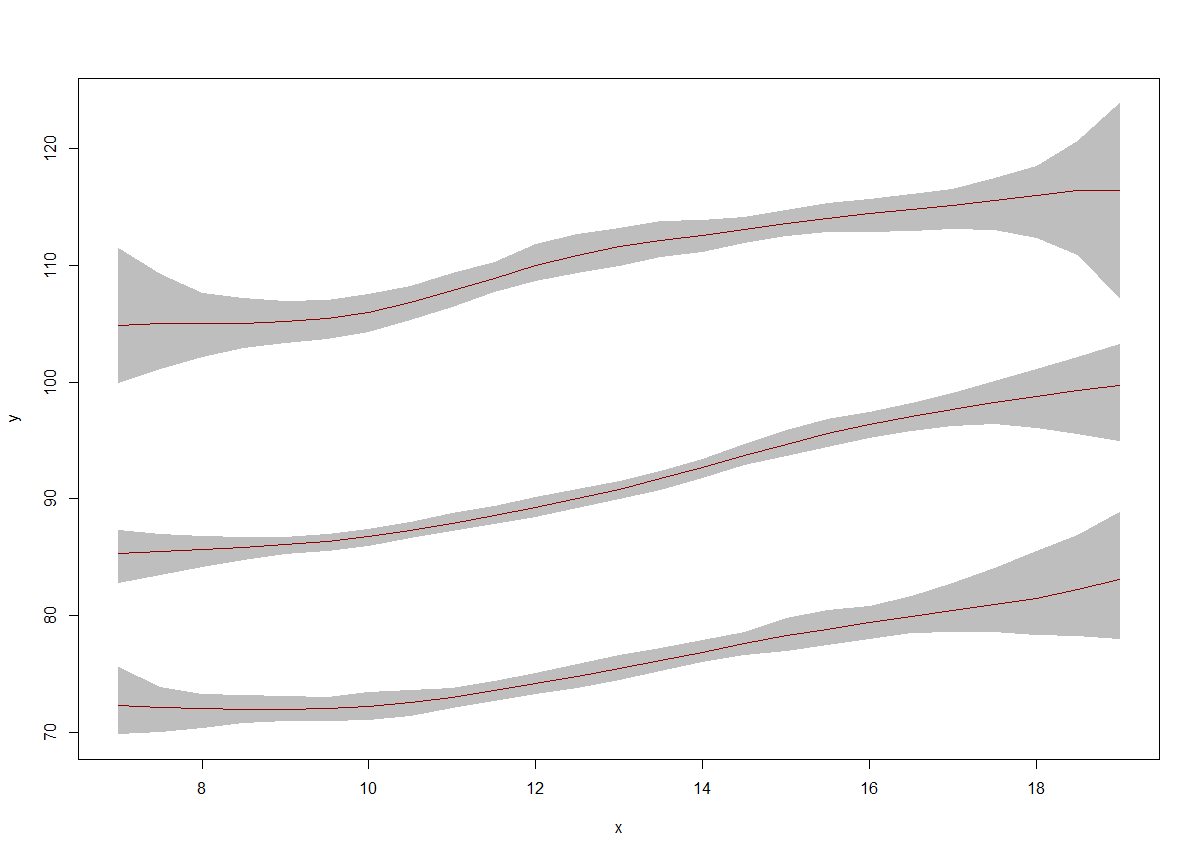


Figure 28S. Nomogram analysis for QT: scatterplot.


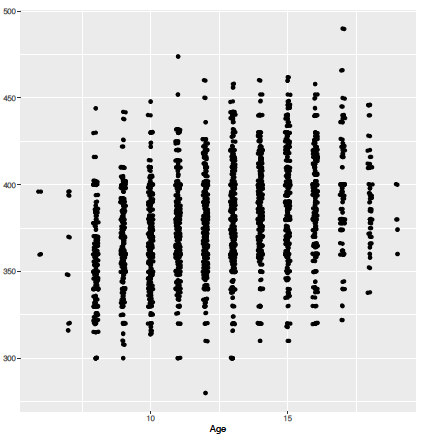


Figure 29S. Nomogram analysis for QT: modeling diagnostics.


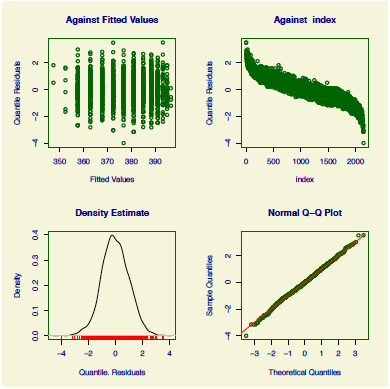


Figure 30S. Nomogram analysis for QT: scatterplot and superimposed centile line plot.


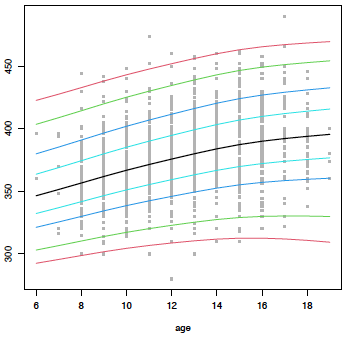


Figure 31S. Nomogram analysis for QT: centile rainbow plot.


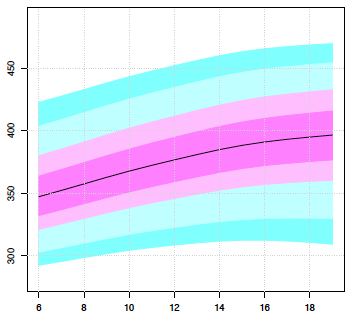


Figure 32S. Nomogram analysis for QT: predicted centile plot.


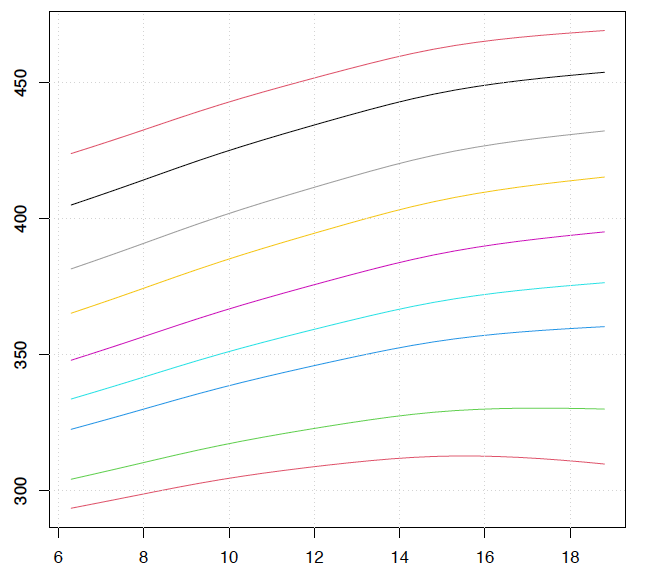


Figure 33S. Nomogram analysis for QT: bootstrap 95% confidence interval for the centiles (2; 50; 98) plot.


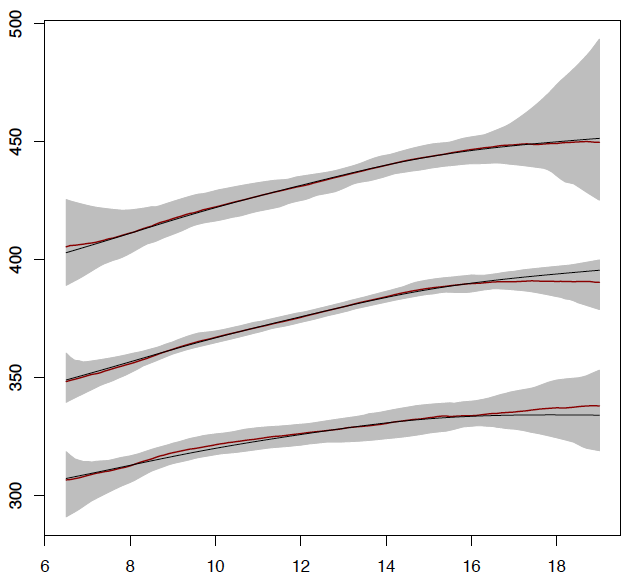


Figure 34S. Nomogram analysis for QTc: scatterplot.


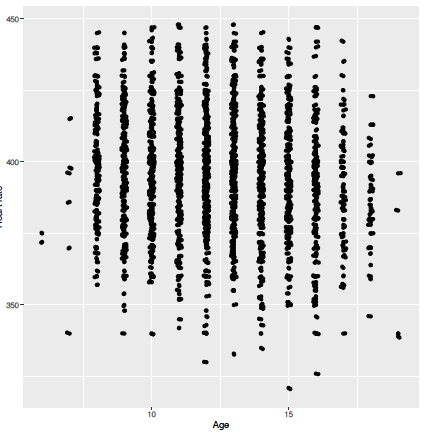


Figure 35S. Nomogram analysis for QTc: modeling diagnostics.


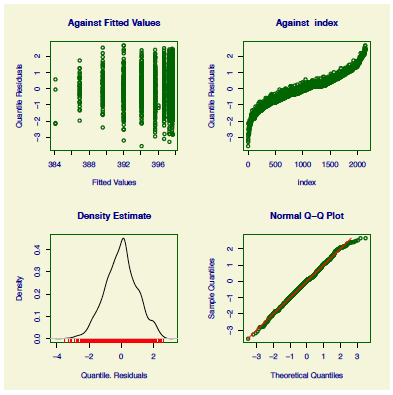


Figure 36S. Nomogram analysis for QTc: scatterplot and superimposed centile line plot.


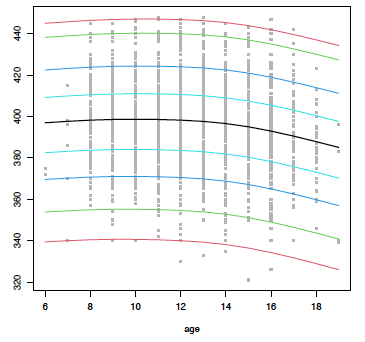


Figure 37S. Nomogram analysis for QTc: centile rainbow plot.


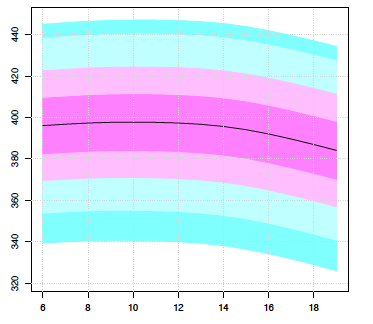


Figure 38S. Nomogram analysis for QTc: predicted centile plot.


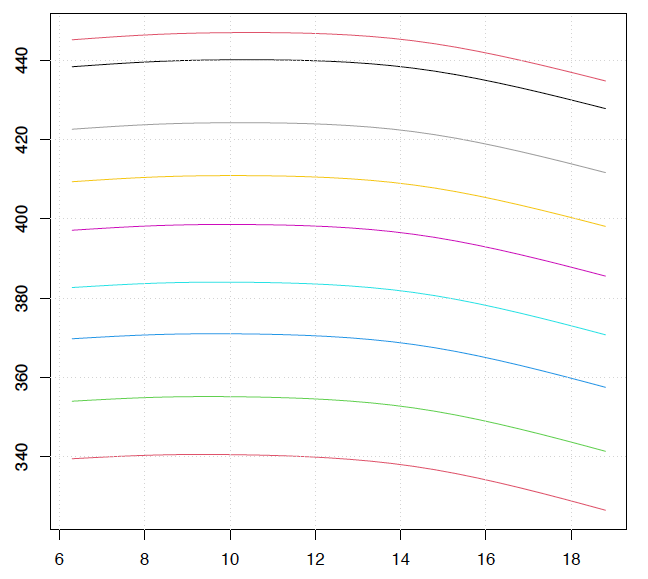


Figure 39S. Nomogram analysis for QTc: bootstrap 95% confidence interval for the centiles (2; 50; 98) plot.


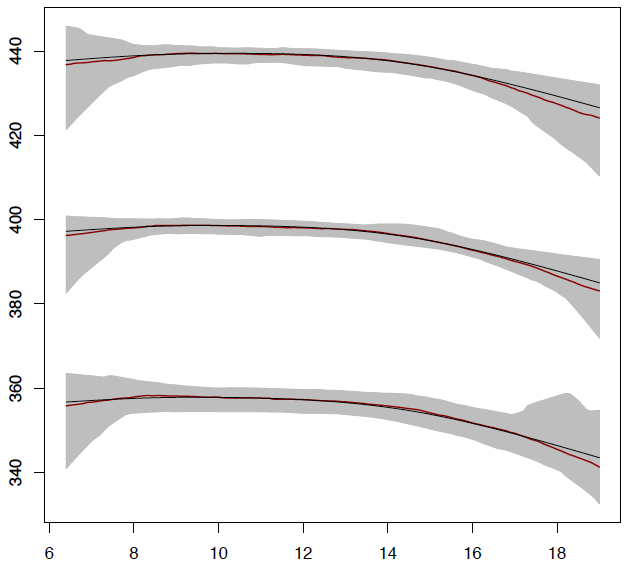


Table 2S. Nomogram analysis for heart rate: age-wise centile table (left panel), and bootstrapped centile table (right panel).

| 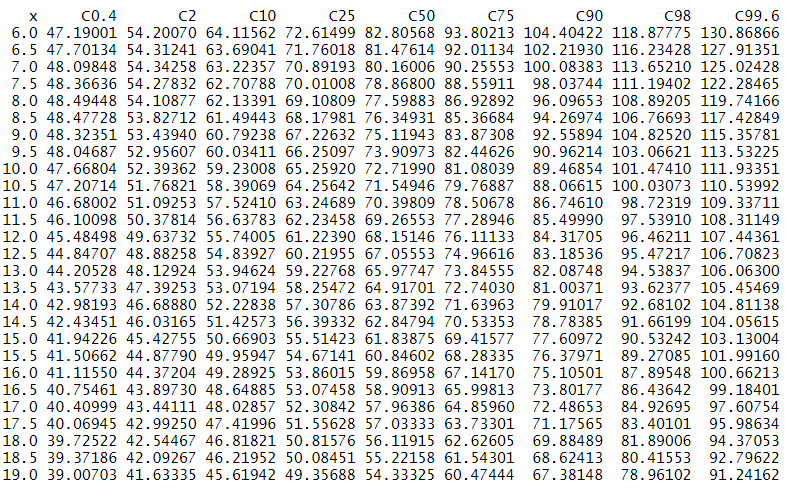 | 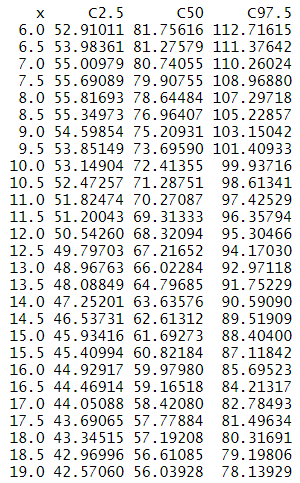 |
| --- | --- |

Table 3S. Nomogram analysis for P wave duration: age-wise centile table (left panel), and bootstrapped centile table (right panel).

| 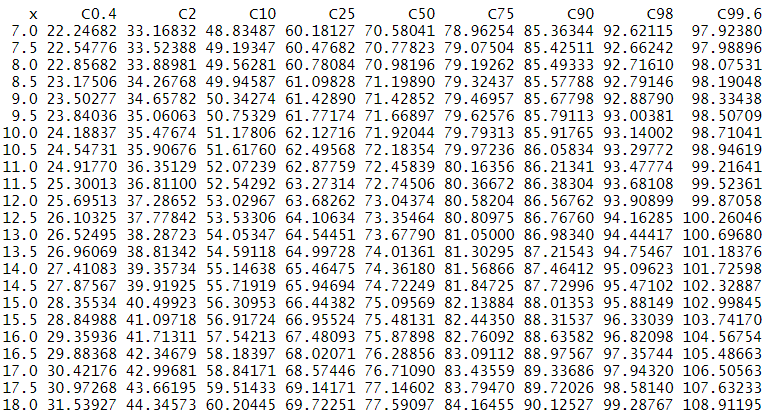 | 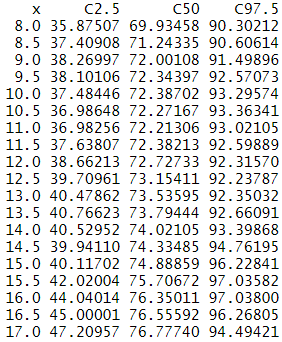 |
| --- | --- |

Table 4S. Nomogram analysis for PR: age-wise centile table (left panel), and bootstrapped centile table (right panel).

| 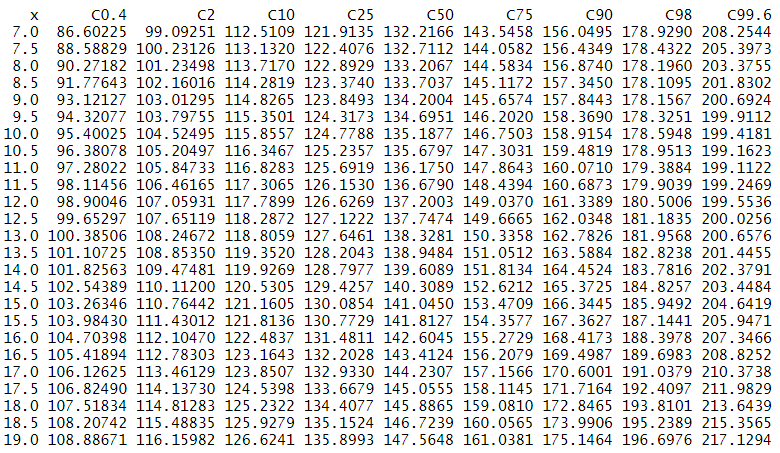 | 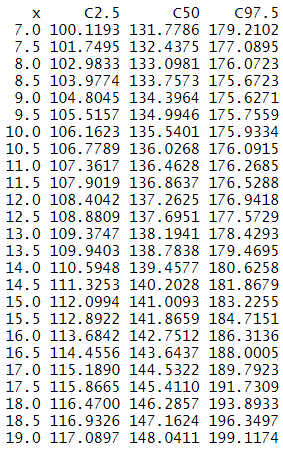 |
| --- | --- |

Table 5S. Nomogram analysis for QRS: age-wise centile table (left panel), and bootstrapped centile table (right panel).

| 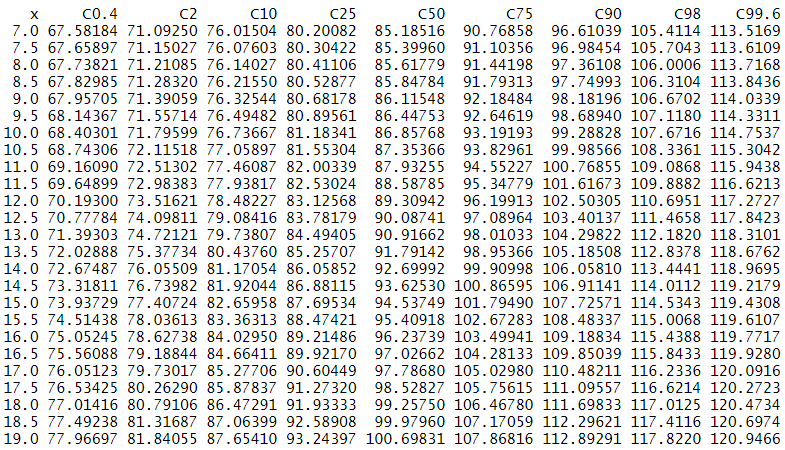 | 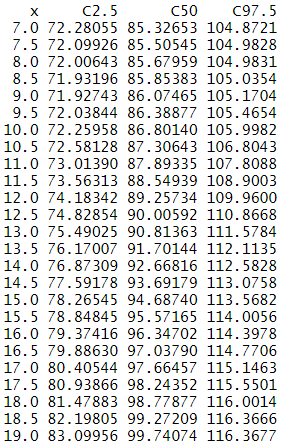 |
| --- | --- |

Table 6S. Nomogram analysis for QT: age-wise centile table (left panel), and bootstrapped centile table (right panel).

| 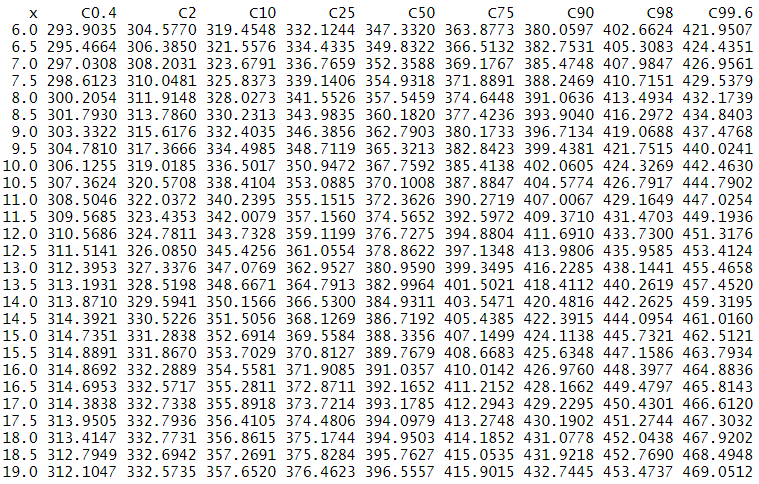 | 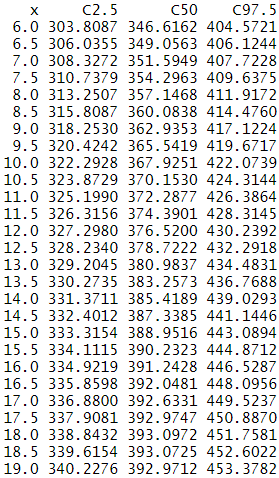 |
| --- | --- |

Table 7S. Nomogram analysis for QTc: age-wise centile table (left panel), and bootstrapped centile table (right panel).

| 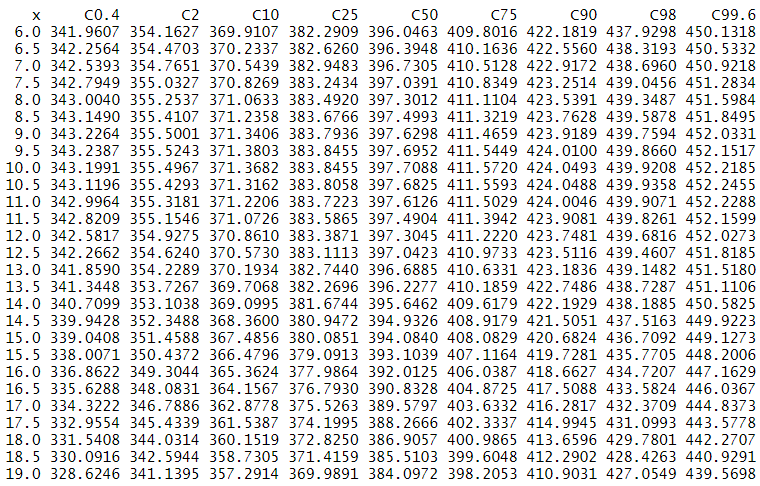 | 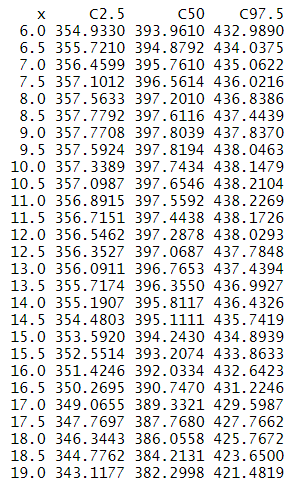 |
| --- | --- |

Figure 40S. Cluster dendrogram highlighting the correlation between individual features and ECG variables.


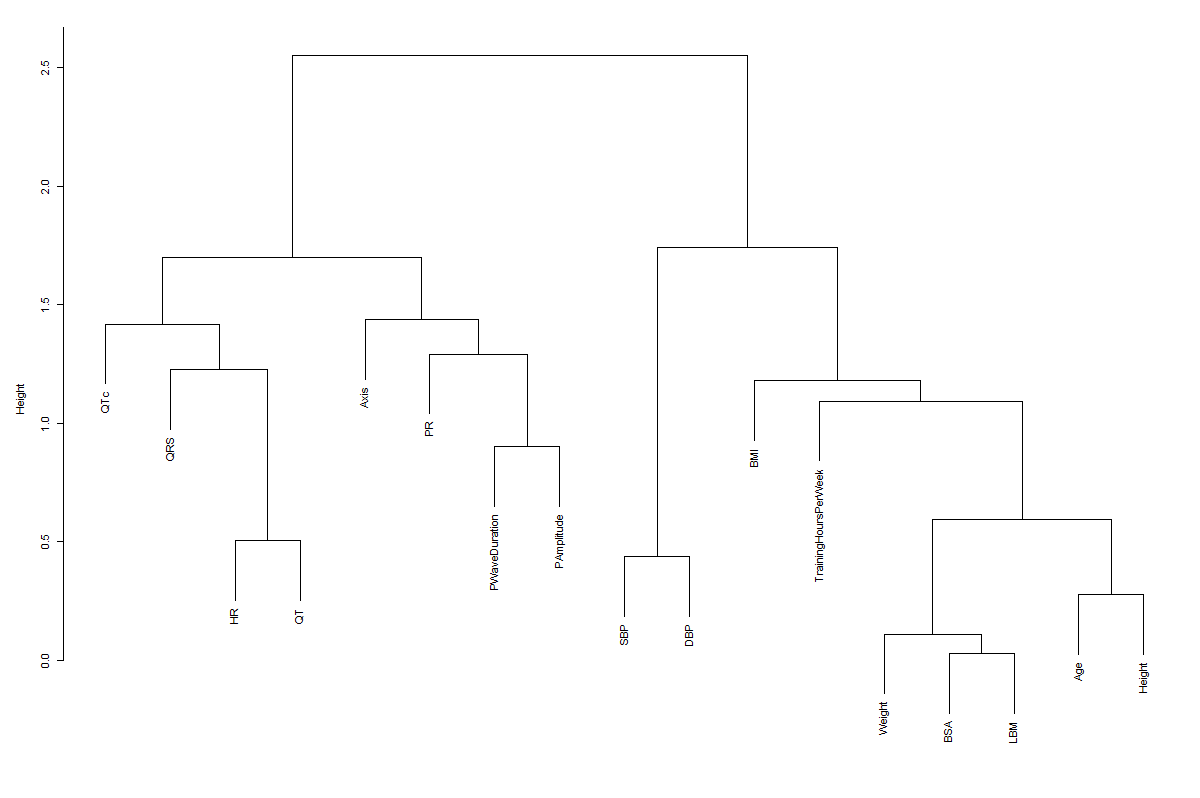

Supplement: Supplementary file 1 [file Data_Sheet_1.docx]
